# How Anaemia Perceptions Shape Dietary Diversity Practice in Pregnancy: A Health Belief Model Study in Tanzania

**DOI:** 10.24248/eahrj.v9i1.826

**Published:** 2025-09-30

**Authors:** Saada Ali Seif

**Affiliations:** a Department of Public Health and Community Nursing, The University of Dodoma, Dodoma, Tanzania

## Abstract

**Background:**

Maternal dietary diversity can significantly impact pregnancy outcomes for both the mother and the fetus. A varied and balanced diet during pregnancy provides crucial nutrients that support the baby's growth and development, as well as the mother's overall health, including being free from maternal anaemia. However, not much is known about the maternal dietary diversity practice in Tanzania. Identifying the magnitude and addressing the possible factors associated with maternal dietary diversity would have a significant contribution to enhancing the nutritional and health status of both the mother and her fetus. The use of the health belief model in this study helped to identify the belief factors influencing the behavioral practice, and this marked the aim of this study.

**Methods:**

This was a community-based analytical cross sectional study that was conducted in Ilala Municipality. It involved 367 pregnant women with gestational ages ranging from 13 to 40 weeks who were selected using a multistage sampling. The data were collected using an interviewer administered structured questionnaire. The data was described using descriptive statistics, and a Pearson correlation test and a linear regression model were used to determine the associations between the outcome variable and its explanatory variables. A standardized beta coefficient, a 95% confidence interval (CI), and a *P* value were reported. A statistically significant variable in the final model was declared at the P value of <.05.

**Results:**

Nearly half of the participants were between the ages of 25 and 34 years (46.4%) and (45.2%) were primigravida. Only (35.1%) of women had good dietary diversity practice with a mean dietary diversity score of 3.97±1.4. The food groups that were mostly consumed were grains and tubers (100%), dark green leafy vegetables (61.3%) and other types of vegetables (53.7%). The majority of pregnant women (84.7%) believed they are at high risk of getting anaemia in any stage of pregnancy. Over 90% of pregnant women believed that anaemia in pregnancy can led to foetus complications, and maternal and foetus death. Substantial proportion of pregnant women reported barriers to access and afford iron rich food. Predicators of good dietary diversity practice for the studied pregnant women include perceived moderate risk of anaemia (Beta = 0.19; CI = 0.2–1.8), and perceived high severity (Beta = −0.09; CI = −0.7 − 0.01), having a higher education level (Beta = 0.8; CI = 0.1–0.8).

**Conclusion:**

Pregnant women are not meeting adequate nutritional requirements. Accessibility and costs are barriers that contribute to low consumption of vitamin A-rich fruits, eggs, nuts, seeds, and dairy products. Higher education level, perceiving the risk, and severity of anaemia during pregnancy are the predictors of good dietary diversity practice. We recommend a tailored intervention, highlighting the risk and severity of pregnancy-related anemia and the benefits of dietary diversification with locally available iron sources.

## BACKGROUND

Dietary diversity is the number of different foods or food groups consumed over a given reference period.^1^ Dietary diversification is one of the best strategies highly recommended to pregnant women,^2^ because during this period, women have higher micronutrient needs and are at risk of micronutrient deficiencies, including iron deficiency, which is a primary cause of anemia.^3^ Severe anemia can place the mother and the baby in danger through an increased risk of blood loss during labour and an increased risk of preterm delivery, low birth weight, and perinatal mortality.^3^ In Tanzania, the prevalence of anaemia among pregnant women aged 15 to 49 years is 55.7%.^4^ This prevalence is 38% higher than the WHO threshold of 40%,^5^ and may indicates a widespread deficiency of micronutrients.

Dietary diversity during pregnancy promotes the health status of the mother and her fetus by ensuring the delivery of adequate amounts of selected micronutrients to meet the increased nutrient demands, including iron, and it is an indicators of nutritional adequacy.^6^ Studies show that dietary diversity of five or more food groups during pregnancy improves iron, folate, and vitamin B12 status, which are key nutrients for preventing maternal anemia and supporting healthy fetal development.^7,8^ However, evidences show that only 32% of women in Tanzania meet the minimum dietary diversity.^4^ Maternal inadequate dietary diversity contributed to 7% of the global disease burden,^9,10^ and one million neonates die in the first week of life, linked with increasing trends in maternal anemia.^11^

In limited resource environments across the globe, some dominant plant-based staple foods and diets lack vegetables, fruits, and animal-source foods.^12^ The African and Asian women reported consuming more cereal-based foods compared to those from the Caribbean, Central, and South America, who consumed more fat, protein, calories, and carbohydrates.^13^ The mean dietary diversity score in East African countries is low, and there is notable variation across different regions. The average dietary diversity mean score in Ethiopia is 3.75±1.35,^14,15^ and 6.84±1.46, in Kenya.^16^ Cereal foods were the most popular food group consumed by pregnant women, followed by other vegetables, plant-based foods from nuts and pulses, other vitamin A-rich fruits and vegetables, milk, and milk products.^14,16,17^ The main identified factors for poor dietary diversity in most African countries were socioeconomic and demographic factors, food insecurity, nutrition awareness, and food taboos during pregnancy.^11,15,17,18^ However, not much is known about the dietary diversity in Tanzania. Identifying the magnitude and addressing the possible factors associated with maternal dietary diversity would have a significant contribution to enhancing the nutritional and health status of both the mother and her fetus.

The use of the Health Belief Model (HBM) in this study will help to identify the beliefs factors influencing behavioral practice. This information is very critical to designing evidence-based, appropriate long-term nutritional intervention strategies to improve the maternal dietary diversity practice, which could address the problem of maternal anemia. Therefore, this study aimed to determine the magnitude of dietary diversity and its associated factors among pregnant women using an HBM in Ilala municipality, Dar-es-Salaam, Tanzania.

### The Health Belief Model

This study adopted the Health Belief Model of behaviour change (HBM),^19^ which guided the identification of predictors of maternal dietary diversity. The model was initially conceived by social psychologists in the public health arena as a way of predicting who would utilize screening tests and/or vaccinations.^20^ The health belief model assumes that one's action in the prevention of illness depends upon the individual's perceptions that they are personally vulnerable to the condition and that perhaps, the consequences of that illness will be serious.^19^ The model further assumes that an individual acts only contrary to the illness if the precautionary behaviour effectively prevents the situation, and the step taken to reduce the risk of the condition exceeds the costs of taking action.

The model is explained further through its five assumptions: (i) Perceived susceptibility, implies that the likelihood of individuals engaging in protective behaviour (e.g., dietary diversification) against illness (such as anaemia) depends on how a person perceives their vulnerability or risk of anaemia. Such individuals may improve their dietary diversity to prevent themselves from suffering from anaemia. (ii) Perceived severity, which refers to how severe the individual believes the effect of developing the condition is. An action of having dietary diversity is taken to prevent anaemia if She believes that anaemia would have consequences for her and her foetus's health (iii) Perceived benefit, which entails that, to change a behaviour depends on the belief that a precautionary behaviour taken is helpful, and this brings the motivation to change. Thus, a woman may strive to have maternal dietary diversity on the belief that this behaviour helps prevent anaemia during pregnancy. (iv) Perceived barriers which imply the inability to adopt a health behaviour change due to feelings on the obstacles to performing a recommended health action, which leads to cost/benefit analysis. Thus, a woman who perceives that some types of food are not accessible, not available, and not affordable will have poor dietary diversity practices. However, belief alone is not enough to motivate an individual to act. Acting involves (v) Cues to action, which are the stimulus needed to trigger the decision-making process to accept a recommended health action. In this study, it is the support from the husband and other family members of the pregnant woman.^20^

## METHODS

### Study Design and Setting

This was a community-based study using an analytical cross-sectional design conducted in Ilala municipality, which is among of the 5 municipalities of Dar es Salaam city in Tanzania. There are 159 streets and 36 Wards in Ilala, with a total of 23,787 residents as per the national population and housing census of 2022, of which 11,929 were women.^21^ The selection of this Municipal is because the statistics show that anemia had a fluctuating frequency among all pregnant women who visited antenatal clinics in the municipality for three consecutive years, 1.7% in 2018, 2.0% in 2019, and 1.5% in 2020.^22^ This raised a concern to explore more about the problem in this area. The major food crops grown within the municipality include cassava, sweet potatoes, paddy, maize, and cowpeas. The residents of the Ilala municipality regard vegetables such as amaranthus, chinese cabbage, eggplants, okra, kale, and leek, and fruits like citrus, passion fruit, papayas, pineapples, mangoes, cucumbers, and cashew nuts; as cash crops.^23^

### Study Population and Participants

The study population was all pregnant women aged between 18 and 49 years, living in Ilala municipality. The study included only those with gestation ages from 13 to 40 weeks, and agreed to give consent to participate, while those who were ill were excluded.

### Sample Size Estimation

The sample size was 367 participants, which was calculated using a Cochran formula for a cross-sectional study as described elsewhere.^24^

### Sampling Procedure

A three-stage probability sampling technique was used, whereby in the first stage, 12 out of 36 wards were selected using a simple random sampling method. The second stage involved the random selection of 2 streets from each ward, and in the third stage, a house-to-house survey was conducted with the help of a community health worker and a 10-house leader to identify pregnant women who were present (Table 1).

**TABLE 1:** Sampling Procedures

Unit	Selection method
Wards (1st Stage)	Twelve wards out of 36 were selected by simple random sampling methods by lottery method with replacement
Streets (2nd Stage)	Two streets were randomly selected in each of the twelve wards to make a total of 24 streets, which were selected by using a simple random sampling by lottery method with replacement
Household (3rd Stage)	All households with eligible participants were identified with the help of the community health workers (CHWs). If household had more than one eligible individuals, all of them were recruited.

### Data Collection Methods and Tools

An interviewer-administered questionnaire was used to collect data from pregnant women in house to house for four weeks in April 2022. The principal investigator collected the data and two trained research assistants who are nurses by profession. The social-demographic and obstetric characteristics were collected using a structured, pre-tested tool adopted from Gibore et al.^25^ and Stephen et al.^26^ Data on dietary diversity recall was collected using a modified tool of the Minimum Dietary Diversity for Women (MDD-W) questionnaire developed by the Food and Agriculture Organization (FAO).^27^ The tool has a total of 10 food groups, but the examples of foods in each group, which are commonly available in our settings, were added. The tool for assessing the constructs of HBM related to maternal diet was developed from the literature.^28^

### Measurement of variables

#### Dependent Variable

**Dietary diversity:** This variable was assessed by 10 items and measured in a binary scale (yes/no) on whether a type of food group was consumed in the past 24 hours. These include food made from the (i) grains, roots, tubers, and plantains (e.g. bread, rice, maize); (ii) pulses and legumes (e.g. beans, peas, and lentils); (iii) nuts and seeds (e.g. peanuts); (iv) dairy products (e.g. fresh milk, butter); (v) meat (red meat, poultry, and fish); (vi) eggs (of hen or duck); (vii) dark green vegetables (e.g. spinach); (viii) other fruits (e.g. banana, orange), (ix) vegetables high in vitamin A (e.g. carrot, papaya); and (x) other vegetables (cabbage, onion). The ‘yes’ response is given a score of ‘1’, and a ‘no’ response is scored ‘0’. The total score is 10, if a woman gets a score of 5 points or above (i.e. consumed five food groups or more) is considered to have good dietary diversity, and if she scores less than 5 is considered to have poor dietary diversity. The mean Dietary Diversity Score (DDS) was then computed by summing up all the individual dietary diversity scores and dividing them by the number of respondents.^29^

#### Independent Variables

**HBM Constructs measurements:** This was measured by using Likert scale questions of 5-point. The degree of agreement about the perception of anaemia was scored 1= strongly disagree to 5= strongly agree. The higher scores show positive perceptions. The *perceived susceptibility* was assessed by 3 items and measured in a Likert scale, on whether the pregnant woman perceives that she is susceptible to anaemia due to pregnancy. The score ranges from 3–15 points, where a score of 3–6 is low, 7–9 is moderate, and 10–15 is high perceived anaemia in pregnancy susceptibility. *The perceived severity* was assessed by 5 items and measured in a Likert scale, on whether the pregnant woman perceives that the effect of anaemia is so severe for the woman and the foetus. The score ranges from 5–25 points, where a score of 5–10 is low, 11–15 is moderate, and 16–25 is high perceived anaemia severity. *The perceived benefit* was assessed by 3 items in a Likert scale on whether the pregnant woman perceives that good maternal dietary diversity practice is beneficial in preventing anaemia. The score ranges from 3–15 points, where a score of 3–6 is low, 7–9 is moderate, and 10–15 is high perceived benefit of maternal dietary diversity practice. *The perceived barrier* was assessed by 5 items measured in a Likert scale on whether the pregnant woman perceives that there are barriers hindering her from having good dietary diversity practice. The score ranges from 5–25 points, where a score of 5–10 is less, 11–15 is moderate, and 16–25 is more perceived dietary diversity practice barriers.

**The socio-demographic characteristics:** Nine items were assessed: religion, marital status, occupation, income level, level of education, age, family size, spouse occupation, and having spouse support on diet.

**Obstetric characteristics: ** Eleven items were assessed: gravidity, pregnancy interval, age of pregnancy, parity, number of ANC visits, screening for Hb, HIV, and malaria during ANC, and whether de-worming, IPT or ITNs, or FEFO were given during ANC.

### Data processing and analysis

SPSS version 25 was used to analyse the data. Data were initially cleaned, checked for normality, and organized before being analysed. The data were described using descriptive statistics (frequency distributions and percentages), and inferential statistics (a Pearson correlation test and a linear regression model) were used to explore the associations between the outcome variable (dietary diversity score) and its explanatory variables (the HBM constructs and demographic characteristics). A standardized beta coefficient, a 95% confidence interval (CI), and a p-value were reported. A statistically significant variable in the final model was declared when the *P* value was <.05.

### Ethical considerations

Permission to conduct this study and ethical clearance, and a research approval letter were obtained from the University of Dodoma Research and Publication Office with the reference number MA.84/261/02/A/19. Authorization to conduct the study in Ilala Municipal and the selected wards was obtained from the Ilala Urban District director. Human rights, privacy, and confidentiality were considered in this study. The research objectives, risks, and benefits of the study were explained well to the participants. Verbal and written consent was obtained from the participants, and the questionnaires were answered voluntarily.

## RESULTS

### Socio-demographic Characteristics of the Participants

In this study, the respondent's minimum age was 17 years, and the maximum age was 42 years, while the predominant age group was 25–34 years, 170 (46.4%). The majority, 274 (74.7%), were married 213 (58.0%) and had a secondary education level, 184 (50.1%) had an average monthly income of less than or equal to 100,000 TZS, and majority, 311 (84.7%) reported to have a diet support from a spouse (Table 2).

**TABLE 2:** Socio-demographic Characteristics of the Participants (N=367)

Variable	Frequency (n)	Percent (%)
Maternal Age (in years)		
15–24	137	37.3
25–34	170	46.4
≥35	60	16.3
Marital status		
Single	93	25.3
Married	274	74.7
Education Level		
Primary level	91	24.8
Secondary level	213	58.0
College/University	63	17.2
Maternal Occupation		
Unemployed	180	49.0
Peasant	10	2.7
Employed	30	8.2
Businesswoman	147	40.1
Average monthly income (in Tshs)		
Lower than 100,000/=	184	50.1
100,000–200,000/=	90	24.5
Higher than 200,000/=	93	25.3
Family Size		
1–2	170	46.3
3–4	127	34.6
≥5	70	19.1
Husband/Spouse's Education Level		
Never attended school	4	1.1
Primary level	78	21.3
Secondary level	198	54.0
College/University	87	23.7
Husband/Spouse occupation		
Unemployed	39	10.6
Employed	214	58.3
Businesswoman	108	29.4
Peasant	6	1.6
Husband/Spouse support on diet		
Yes	311	84.7
No	56	15.3

### Obstetric Characteristics and ANC Preventive Services Provided to the Participants

The highest proportion of participants, 309 (84.2%), were in their third trimester, and the majority, 153 (76.1%), had an inter-pregnancy interval of less than 24 months, and 166 (45.2%) were primigravida. Half of the participants, 192 (52.3%) reported 2–3 ANC visits, and the majority, 357 (97.3%) had been taking an iron supplement (FEFO) (Table 3).

**TABLE 3:** Obstetric Characteristics of the Participants (N=367)

Variable	Frequency (n)	Percentage (%)
Gravidity		
Primigravida	166	45.2
Multigravida	99	27.0
Grand multipara	102	27.8
Inter-pregnancy interval		
A short interval of fewer than 24 months	153	76.1
Long intervals of more than 36 months	48	23.9
Trimester of the pregnancy		
Second 13 – 24 weeks	58	15.8
Third 25 – 40 weeks	309	84.2
Experienced vomiting during the index pregnancy		
Yes	312	85.0
No	55	15
Appetite		
Good	204	55.6
Normal	148	40.3
Poor	15	4.1
Number of ANC visits		
One visit	3	0.8
2–3 visit	192	52.3
4 and above	172	46.9
Experienced PICA habits		
Yes	9	2.5
No	358	97.5
Received IPT during ANC visits		
Yes	339	92.4
No	28	7.6
Received ITN during ANC visits		
Yes	345	94.0
No	22	6.0
Received de-worming tablets during ANC		
Yes	297	80.9
No	70	19.1
Use Iron supplement (FEF0) on index pregnancy		
Yes	357	97.3
No	1 0	2.7
Used Multivitamins during the index pregnancy		
Yes	24	6.5
No	343	93.5
Did you screen for malaria during the index pregnancy		
Yes	346	94.3
No	21	5.7

### Dietary Diversity Practice

#### Types of food groups consumed

Table 4 shows the dietary intake among pregnant women during the past 24 hours before the interview. The food groups that were mostly consumed were grains and tubers, 367 (100%) and legumes, 247 (67.3%). The least majority consumed dark green leafy vegetables, 225 (61.3%), and other types of vegetables, 197 (53.7%). The food groups that were consumed less were other fruits (not rich in vitamin A), 41 (11.2%), and eggs, 29 (7.9%).

**TABLE 4:** Frequency Distribution of Types of Food Groups Consumed (N=367)

Food Group	Intake	
	Yes (%)	No (%)
Grains and Tubers	367 (100)	0 (0.0)
Legumes	247 (67.3)	120 (32.7)
Dark green leafy vegetables	225 (61.3)	142 (38.7)
Other Vegetables	197 (53.7)	170 (46.3)
Meat Poultry and Fish	130 (35.3)	237 (64.6)
Vitamin-A Rich Fruits and Vegetables	100 (27.2)	267 (72.8)
Dairy Milk and Products	63 (17.2)	304 (82.8)
Nut and Seeds	60 (16.3)	307 (83.7)
Other fruits (not rich in vitamin A)	41 (11.2)	326 (88.8)
Eggs	29 (7.9)	338 (92.1)

### Minimum Dietary Diversity and Dietary Diversity Score

The mean dietary diversity score was 3.97±1.4. The minimum number of food groups consumed was 2, and the maximum was 8. The highest proportion of participants, 207 (56.4%), consumed 3 food groups while the lowest proportion, 4 (1.1%) consumed 8 food groups in the past 24 hours prior to the interview (Table 5) Furthermore, 129 (35.1%) of studied pregnant women had consumed at least 5 food groups in the past 24 hours prior to the interview, and hence were termed as having good dietary diversity.

**TABLE 5:** Dietary Diversity Score (N=367)

Number of food groups	Frequency (n)	Percent (%)
1	0	0.0
2	19	5.2
3	207	56.4
4	12	3.3
5	50	13.6
6	54	14.7
7	21	5.7
8	4	1.1

### Frequency Distribution of Constructs of the Health Belief Model Related to Dietary Diversity

**Perceived Susceptibility: ** The results showed that the slight majority of the participants agreed that they were at risk of getting anaemia during the second trimester, 237 (64.6%), 231 (62.9%) believed they are at risk during the first trimester, and 225 (61.3 %) during the third trimester (Table 6).

**TABLE 6:** Frequency Distribution of Constructs of Health Belief Model Related to Dietary Diversity (N=367)

Variable	SD	D	N	A	SA
	n (%)	n (%)	n (%)	n (%)	N (%)
Perceived Susceptibility					
I believe I am at risk of getting anaemia during the first trimester	13 (3.5)	]32 (8.7)	27 (7.4)	231 (62.9)	64 (17.4)
I believe I am at risk of getting anaemia during the second trimester.	12 (3.3)	25 (6.8)	21 (5.7)	237 (64.6)	72 (19.6)
I believe I am at risk of getting anaemia during the third trimester	24 (6.5)	24 (6.5)	20 (5.4)	225 (61.3)	74 (20.2)
Perceived Severity					
I believe that anaemia during pregnancy can result in abortion, intra-uterine growth restriction, and intra-uterine foetal death	10 (2.7)	7 (1.9)	42 (11.4)	193 (52.6)	115 (31.3)
I believe that anaemia can lead to maternal death during pregnancy	11 (3.0)	1 (0.3)	23 (6.3)	218 (59.4)	114 (31.1)
I believe that anaemia in pregnancy can affect the family economically	12 (3.3)	9 (2.5)	40 (10.8)	238 (64.9)	68 (18.5)
I believe that when a woman gets anaemia it can result in complications for a foetus and new-born baby.	11 (3.0)	8 (2.2)	34 (9.3)	233 (63.5)	81 (22.1)
Perceived Benefits					
I believe that taking enough animal food can prevent developing anaemia during pregnancy	13 (3.5)	19 (5.2)	67 (18.3)	221 (60.2)	47 (12.8)
I believe that taking enough fruits (papaya, oranges, and mangoes) can prevent developing anaemia during pregnancy	8 (2.2)	17 (4.6)	23 (6.3)	248 (67.6)	71 (19.3)
I believe that taking enough green vegetable food (spinach and green leaves) can prevent developing anaemia during pregnancy	9 (2.5)	10 (2.7)	3 (0.8)	233 (63.5)	112 (30.5)
Perceived Barriers					
I believe that it is too far to get food rich in iron to me (accessibility)	118 (32.2)	206 (56.1)	22 (6.0)	17 (4.6)	4 (1.1)
I believe that I cannot afford to buy food rich in iron (affordability)	78 (21.3)	183 (49.9)	19 (5.2)	82 (22.3)	5 (1.4)
I believe that animal product foods are not available in my location (availability)	120 (32.7)	195 (53.1)	17 (4.6)	34 (9.3)	1 (0.3)
I believe that fruits are not available in my location	128 (34.9)	190 (51.8)	2 (0.5)	38 (10.4)	9 (2.5)
I believe that green vegetable foods are not available in my location	129 (35.1)	206 (56.1)	1 (0.3)	24 (6.5)	7 (1.9)

**Perceived Severity:** The highest proportion of participants agreed that anaemia in pregnancy can affect the family economically, 238 (64.9%), and anaemia can result in complications for the foetus and a new-born baby, 233 (63.5%), while the least proportion agreed that anaemia during pregnancy can result in abortion, 193 (52.6%), (Table 6).

**Perceived Benefits:** The results revealed that the highest proportion of participants agreed that taking enough fruits (papaya, oranges, and mangos), 248 (67.6%), and taking enough green vegetable food (spinach and green leaves), 233 (63.5), could prevent anaemia during pregnancy, and 221 (60.2%) agreed that, taking enough animal food could prevent developing anaemia during pregnancy (Table 6).

**Perceived Barriers:** The findings showed that a low proportion of participants agreed that it was too far to get food rich in iron 118 (32.2%), and they cannot afford to buy food rich in iron, 78 (21.3%), and 128 (34.9%) agreed that fruits were not available in their location area (Table 6).

### Categorization of HBM constructs related to dietary diversity

Results of this study showed that about 311 (84.7%), had a high perceived anaemia in pregnancy susceptibility, 349 (95.1%), had a high perceived anaemia severity, 340 (92.6%), had a high perceived benefit of maternal dietary diversity practice in prevention of anaemia, and 265 (72.2%) had less perceived dietary diversity practice barriers.

### Predictors of Dietary Diversity Among Pregnant Women

In determining the predictors of dietary diversity, the dietary diversity score was the dependent variable, and the independent variables were the demographic variables and the HBM constructs. Initially, a one-way ANOVA and a bivariate correlation were run to determine the correlation between variables, and those with a *P* value of 0.2 and below were included in the linear regression model, where all categorical variables were dummy coded. The results showed that the dietary diversity score was significantly correlated with perceiving the susceptibility (*P*=.01), education level (*P*<.001), occupation (*P*=.03), and husband/spouse support (*P*=.03) (Table 7).

**TABLE 7:** Bivariate Correlation Between Dietary Diversity Score and HBM Constructs and Demographic Variables of Pregnant Women (N=367)

Variable	Dietary diversity Mean score (SD)	*P*-value
Perceived Susceptibility		
High	3.97 (1.4)	.006
Moderate	4.53 (1.8)	
Low	3.25 (1.1)	
Perceived Benefit		
High	3.99 (1.4)	.3
Moderate	3.33 (1.0)	
Low	4.06 (1.4)	
Perceived Severity		
High	3.95 (1.4)	.14
Moderate	3.71 (1.0)	
Low	4.8 (1.7)	
Perceived Barriers		
High	3.9 (1.4)	<.001
Moderate	3.8 (1.3)	
Low	4.0 (1.5)	
Education Level		
Primary level	3.4 (1.2)	<.001
Secondary level	3.99 (1.4)	
College/University	4.69 (1.4)	
Maternal Occupation		
Unemployed	3.8 (4.1)	.03
Peasant	3.1 (0.8)	
Employed	4.1 (1.4)	
Businesswoman	4.1 (1.4)	
Husband/spouse support on diet		
Yes	4.0 (1.4)	.03
No	3.5 (1.3)	

### Multiple Linear Regression Model Results

In the linear regression model, the results showed that a unit increase in the degree of perceived anaemia susceptibility increases the degree of dietary diversity score by 0.19 (Beta = 0.19; CI = 0.2–1.8; *P*=.01). Moreover, a unit increase in the degree of perceived severity of anaemia effect decreases the dietary diversity score by 0.09 (Beta = −0.09; CI =−0.7 –(−)0.01; *P*=.04). Furthermore, a unit increase in the education level of a woman from primary level to secondary level, increases the dietary diversity score by 0.89 unit (Beta = 0.8, CI =0.1–0.8; *P*=.003), and the increase in education level from primary to college level, increases the dietary diversity score by 1.6 unit (Beta = 1.6, CI = 0.6–1.6; *P*<.001) (Table 8).

**TABLE 8:** Linear Regression Results of Pregnant Women's Predictors of Dietary Diversity (N=367)

	Unstandardized Beta	Std error coefficient	Standardized Beta	95% C.I.		*p*-*value*
				Lower	Upper	
Perceived Susceptibility						
High	0.47	0.32	0.11	−0.17	1.11	.15
Moderate Low	1.03	405	0.19	0.23	1.83	.01
Low	Ref					
Perceived Severity						
High	−1.03	0.48	−0.09	−1.73	−0.01	.04
Moderate Low	−0.87	0.71	−0.12	−0.24	0.37	.1
Low	Ref					
Education Level						
Primary level	Ref					
Secondary level	0.53	0.18	0.17	0.18	0.89	.003
College/University	1.12	0.24	0.28	0.64	1.61	<.001
Maternal occupation						
Unemployed	Ref					
Employed	0.64	0.32	0.01	−0.58	0.70	.8
Business woman	0.23	0.19	0.07	−0.14	0.70	.2
Peasant	−0.38	0.48	−0.04	−0.14	0.60	.4
Average monthly income	0.65	0.12	0.03	−0.17	0.30	.5
Husband support						
Yes	0.35	0.21	0.08	−0.06	0.77	.09
No	Ref					

## DISCUSSION

Dietary diversity during pregnancy promotes the health status of the mother and her fetus by ensuring the delivery of adequate amounts of selected micronutrients to meet the increased nutrient demands, including iron, which is responsible for the prevention of maternal anemia. Initiatives to address anaemia need evidence of its trends and relevant factors, as they vary from one area to another. This study assessed the dietary diversity and its associated factors among pregnant women, using a health belief model, in Tanzania.

The majority of pregnant women in this study exhibited poor dietary diversification (64.9%). The average dietary diversity score was 3.97±1.4, which is lower than the recommended minimum dietary diversity for women, which is 5.^30^ This suggests nutrient deficiency, which implies that pregnant women in the study area are not acquiring adequate amount of nutrients to support the growing fetus, and thus they are at risk of anemia, low birth weight, preterm birth, and stillbirth.^31^ Thus, policy and program consideration in this area is warranted. Our finding is lower than that found in Kenya (6.84±1.46).^16^ The characteristics of the research participants, which vary depending on where they were recruited, may be the cause of this disparity. The study in Kenya was conducted in a hospital, and it is widely known that people who use ANC services are more likely to be aware of the health concerns associated with pregnancy and take preventative measures (such as food diversification), as well as have the financial resources to do so. However, our study was conducted in a community where there is a higher chance of recruiting even those who do not attend the ANC as required.

It was noted in this study that pregnant women rarely consumed eggs, nuts, seeds, dairy milk and products, and vitamin A-rich fruits and vegetables, but the vast majority consumed grains and tubers. This result is consistent with most studies in low and middle-income countries, which show that a cereal-based diet was the most popular.^13,15,32,33^ Low-income countries reported relying more heavily on locally produced cereals, roots, tubers, and plantains, which are readily available and affordable, while the supply of higher-value food products, such as meat and dairy products, fruit, and vegetables, only increases with the rise in the level of income.^34^ In addition, studies have reported that animal products are the most popular foods avoided during pregnancy on cultural grounds.^10^ This is the area to focus on in future studies to explore qualitatively the reasons for avoiding animal products during pregnancy. Therefore, the results of the current study imply that the requirement for sufficient energy and key nutrients for pregnant women is suggested as not met.

Additionally, the results of this study show that most pregnant women had a favourable opinion of susceptibility, severity, and benefits, indicating that most of them are aware that they are at risk of developing anaemia due to pregnancy, the consequences of anaemia to maternal and foetal health and the benefits of diet on preventing anaemia. Food types such as animal products, fruits, and vegetables were considered essential in the prevention of anaemia by a majority of women, however, these types of food were the most mentioned to face the accessibility, availability, and affordability problems among most participants. This could explain why the majority of women in this study reported not consuming fruits and animal products. Moreover, women may have developed high perceptions either from public media (radio and television) where there is regular broadcasting of health programs, or from previous experience or family. This is encouraging because perceiving the risk, severity, and benefit is one step ahead of having healthy behaviour, which is dietary diversification in our case.

Looking at the predictors of good dietary diversity, perceiving the risk and perceiving severity were the two constructs that were shown to predict good dietary diversity in this setting, while there was not enough evidence to confirm the association between perceiving the benefits and barriers as suggested by the model. The lack of significant association could have been contributed to the small sample size, the large variation in the groups, or the weakness of the data collection tool. For example, the questions used to assess the benefit focused on asking about the benefit of taking each type of food separately and not the benefit of dietary diversification in the prevention of anaemia. Moreover, the questions to assess the barriers did not ask about the availability, affordability, and accessibility for each type of food group rather, some food was assessed for their availability, and others for their accessibility and affordability. Thus, the results may not reflect the complete barriers to food diversification.

Surprisingly, perceiving the severity was inversely related to dietary diversification. Those who perceived pregnancy complications of anaemia as more severe were less likely to have good dietary diversity practice. This could be due to food taboos often associated with pregnancy, where certain foods are believed to cause birth defects or lead to pregnancy complications. This type of belief is reported in many African societies.^15^ Future studies in Tanzania need to explore more about this matter qualitatively.

The woman's education level was also a statistically significant predictor of good dietary diversity. In this study, highly educated women are more likely than less educated women to have good dietary diversity. It has been well established in the literature that educated women are more likely to exhibit healthy behaviour than uneducated ones.^35^ This result is consistent with what was reported in other studies.^16,17^ Therefore, the government should continue promoting education widely and reducing disparities in access to educational resources to improve future well-being.

### Limitation

It is worth mentioning that we used the perceptions (from HBM constructs) on anaemia and not of Vitamin A deficiency or Folic Acid deficiency, with the assumption that anaemia is considered as a proxy measure of Vitamin A and Folic Acid deficiency. Additionally, the term anaemia is more understandable to women in the community compared to asking about vitamin A or Folic acid deficiency. Importantly, to also worth noting that the questions focused on perceptions of only iron-rich food and not all food groups. Therefore, these results should be interpreted with caution.

## CONCLUSION

Accessibility and costs are barriers that contribute to low consumption of vitamin A-rich fruits and vegetables, eggs, nuts, seeds, and dairy products. Higher education level, perceiving the risk, perceiving the severity of anaemia during pregnancy are the predictors of good dietary diversity practice. Studied pregnant women are not meeting adequate nutrition requirements to support foetal growth, putting them at risk of anemia, low birth weight, preterm birth, and stillbirth. We recommend a tailored intervention, including an awareness campaign, highlighting the risk and severity of pregnancy-related anemia and the benefits of dietary diversification with locally available iron sources (e.g., beans, dark leafy greens). Additionally, the government and other stakeholders should establish support programs to address barriers to a diverse diet and improve access to formal education. Future studies should investigate pre-pregnancy dietary diversity to prevent nutritional deficiencies before conception.
